# Qatar’s National Expanded Metabolic Newborn Screening Program: Incidence and Outcomes

**DOI:** 10.3390/ijns11030050

**Published:** 2025-06-30

**Authors:** Tala Jamaleddin, Karen El-Akouri, Sumaya Abiib, Rola Mitri, Mamatha Ramaswamy, Sara Musa, Rehab Ali, Noora Shahbeck, Hilal Al Rifai, Ghassan Abdoh, Tawfeg Ben-Omran, Osama Y. Al-Dirbashi, Mashael Al-Shafai

**Affiliations:** 1 Department of Biomedical Sciences, College of Health Sciences, QU Health, Qatar University, Doha P.O. Box 2713, Qatar; tj1800900@qu.edu.qa (T.J.);; 2 Division of Genetic and Genomic Medicine, Sidra Medicine, Doha P.O. Box 26999, Qatar; 3 Medical Genetics Department, Hamad Medical Corporation, Doha P.O. Box 3050, Qatar; 4 Department of Lab Medicine and Pathology, Hamad Medical Corporation, Doha P.O. Box 3050, Qatar; 5 Department of Pediatrics and Neonatology, Neonatal Intensive Care Unit, Newborn Screening Unit, Women’s Wellness and Research Center, Hamad Medical Corporation, Doha P.O. Box 3050, Qatar; 6 Weill Cornell Medicine-Qatar, Qatar Foundation—Education City, Doha P.O. Box 24144, Qatar; 7 College of Health and Life Sciences, Hamad Bin Khalifa University, Doha P.O. Box 17666, Qatar; 8 Biomedical Research Center, Qatar University, Doha P.O. Box 2713, Qatar

**Keywords:** newborn screening, Qatar National Newborn Screening Program, Qatar, inborn errors of metabolism, inherited metabolic disorders, incidence

## Abstract

Background: Newborn screening is an essential public health strategy that aims to detect a range of conditions, including inborn errors of metabolism, in neonates shortly after birth. The timely identification is crucial due to the asymptomatic nature of many conditions at birth, but which can lead to significant health complications if left untreated. Through this study, we aimed to investigate the incidence of IEMs screened by the Qatar National Newborn Screening Program. Methods: We retrospectively analyzed a total of 351,223 newborns screened from 2010 to 2023. The incidence for the studied IEMs was calculated and correlated with demographics, consanguinity, and family history. In addition, the diagnostic yield of different tests utilized was assessed. Results: Our study revealed a total of 318 positive cases with IEMs, and a significantly high incidence of 1:1105 for IEMs in Qatar. Classical Homocystinuria was the most frequently detected condition, with a cumulative incidence of 1:6754 live births, linked to the founder variant p. Arg336Cys in the *CBS* gene. Aminoacidopathies were the most prevalent category, followed by fatty acid oxidation disorders, organic acidurias, biotinidase deficiency, and urea cycle disorders. Genetic testing showed a high diagnostic yield of 90%. Of the 60 cases that underwent targeted variant testing, 98% were confirmed, while 90% of the 59 cases tested by single gene testing were confirmed. Conclusions: Our study provides the incidence rates of IEMs in Qatar and novel insights that could facilitate setting up/developing IEM incidence-reducing strategies and improving outcomes for affected newborns and their families.

## 1. Introduction

Newborn screening (NBS) programs represent a cornerstone in public health strategies worldwide, serving as vital tools to detect and address potential health concerns in neonates early on. These programs are designed to identify a spectrum of genetic, metabolic, endocrine, hematological, and other disorders that, if left untreated, could lead to profound health complications or even fatalities [1,2]. By implementing NBS, healthcare systems globally have witnessed significant reductions in the rates of morbidity and mortality associated with these conditions, demonstrating the pivotal role of early intervention in safeguarding infant health [3,4].

The utilization of tandem mass spectrometry (MS/MS) in NBS has significantly enhanced the detection of inborn errors of metabolism (IEMs), allowing for screening of more disorders simultaneously and identifying more cases compared to clinical diagnosis alone [5,6]. This technology has revolutionized the early detection of IEMs, enabling timely interventions to prevent long-term health consequences [5]. In Qatar, the inception of the Qatar National Newborn Screening Program (QNNSP) in 2003 marked a pivotal step towards ensuring the optimal health outcomes of the nation’s newborn population. Since its establishment, the QNNSP has evolved and expanded, currently encompassing screening for a wide range of conditions, including IEMs [1]. Moreover, Qatar’s distinct demographic landscape, characterized by a relatively high prevalence of consanguinity and cultural endogamy, further underscores the critical importance of robust NBS initiatives tailored to address the unique genetic profile of the population [7,8].

Our study aims to investigate the incidence of the IEMs screened in newborns in Qatar over a span of 14 years, from 2010 to 2023. We hypothesize that Qatar may experience a higher incidence of IEMs compared to other countries, attributable to factors such as consanguinity and high rates of cultural endogamy in the population. Additionally, this study examines the association between demographic variables and program findings, investigates genetic findings of IEMs, and evaluates the diagnostic yield of various genetic tests. Understanding the incidence and genetic basis of IEMs is crucial for developing effective management strategies, informing policy decisions, and providing valuable insights for physicians, geneticists, genetic counselors, and dietitians involved in NBS, management, and counseling.

## 2. Materials and Methods

### 2.1. QNNSP Structure, Program Flow, and Pathway

**Figure 1:**
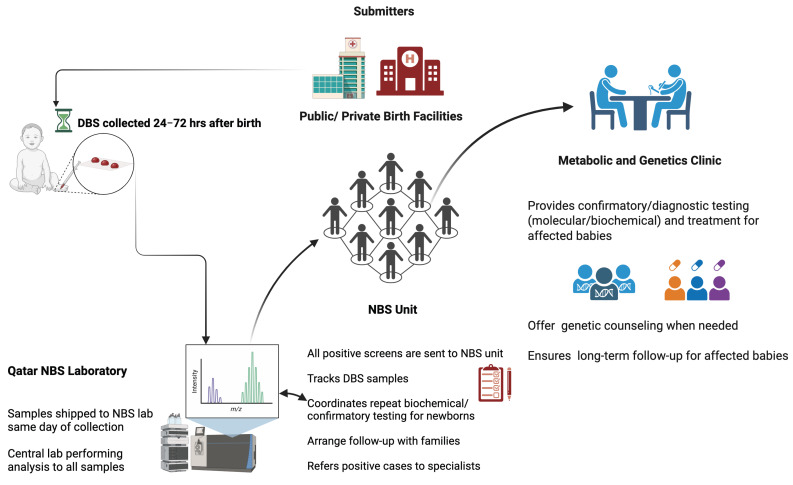
QNNSP Design. Created with BioRender.com.

In Qatar, NBS is not mandated but is universally offered to all newborns free of charge as a standard of care, with a coverage of 98% [9]. The design of our screening program promotes seamless pre-analytical, analytical, and post-analytical processes (see Figure 1). Similarly to other programs, the program encompasses the screening lab, the NBS unit, and the treatment team. Screening results are reported to the NBS unit for retrieval and short-term follow-up and parental education. This is followed by diagnostic testing, patient management, treatment monitoring, and genetic counseling at the metabolic and genetics clinic.

### 2.2. Study Design and Ethical Approval

**Figure 2:**
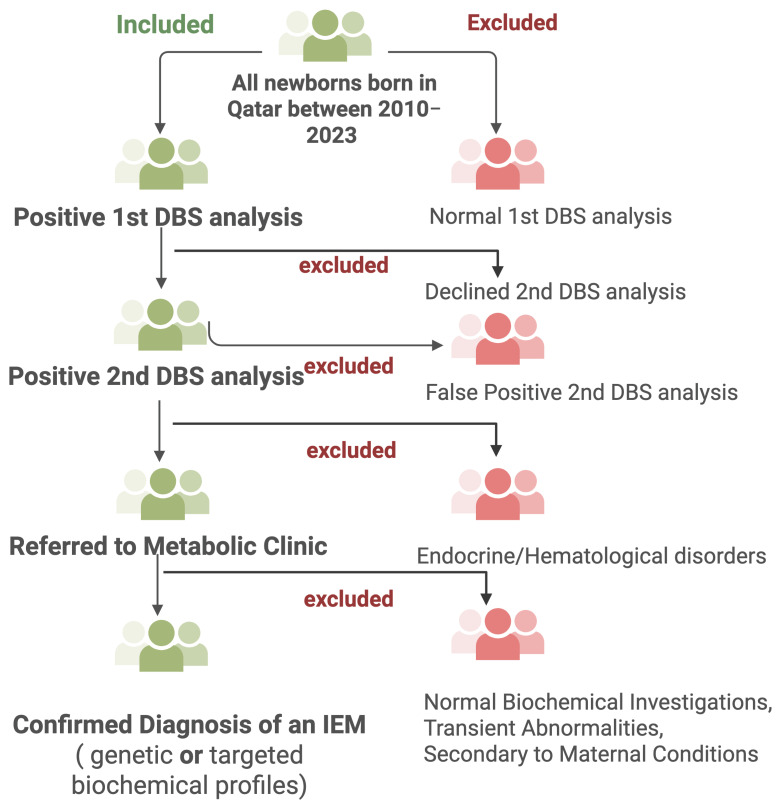
Study design overview. Created with BioRender.com.

This retrospective cohort study was conducted at the NBS unit in the Women Wellness and Research Center (WWRC) and the Metabolic and Clinical Genetics Department at HMC in Qatar. The project was approved by the Medical Research Center at Hamad medical Corporation (HMC) and by the WWRC under the protocol number MRC-01-23-580. Additionally, it was approved by the Institutional Review Board (IRB) of Qatar University under the reference number QU-IRB 010/2024-E. The study involved a filtration process and chart review of newborns who had undergone NBS and were biochemically suspected of having an IEM between 2010 and 2023, using HMC electronic medical records and data from the QNNSP. Figure 2 summarizes the screening workflow, including inclusion and exclusion criteria, as well as the steps leading to a confirmed diagnosis of IEMs.

### 2.3. Study Participants and Data Collection

**Figure 3:**
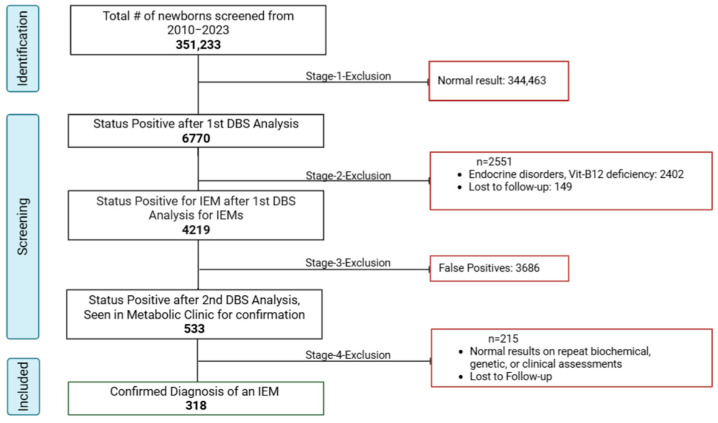
Screening and inclusion of eligible cases.

After applying the inclusion/exclusion criteria, 318 positive cases were included in the analysis. The participant selection process is summarized in Figure 3. The data were extracted from computerized and non-computerized medical records: demographic information (age, gender, ethnicity, consanguinity) and clinical characteristics data (preliminary and secondary diagnoses, familial history, biochemical tests outcomes). If genetic testing was performed, the following data of the results were collected: identified genes, variants, variant classification as per the American College of Medical Genetics and Genomics (ACMG) guidelines, associated genetic condition, inheritance pattern, and genotype–phenotype correlations.

Identified IEMs were categorized according to the metabolic pathway involved in aminoacidopathies, organic acidemias, fatty acid oxidation disorders, urea cycle disorders, galactosemias, and biotinidase deficiency. The purpose was to study the association between these disease groups and factors like ethnicity, consanguinity, family history, and genetic testing.

### 2.4. Incidence Calculation for Identified IEMs

The incidence was calculated using the incidence rate formula that determined the rate at which new cases of IEMs were identified among newborns within Qatar Newborn Population over a 14-year period. The general equation on which our calculation of the incidence rate was based is as follows [10]:

Incidence Rate=(Number of New Cases / Total Population at Risk)×Multiplier

In this study, the total population at risk was defined as the number of newborns who underwent screening through the national NBS program. These data were obtained directly from the NBS unit at the WWRC, which manages the program at the national level. The following three equations were used to present the incidence data, cumulative incidence, annual incidence, and cumulative incidence ratio [10], as shown below:

-
Cumulative Incidence per 100,000=(Number of cases/Population at risk)×100,000

-
Annual Incidence per 100,000=(Cumulative Incidence per 100,000/Number of years (14))

-
1 in X Cumulative Incidence=1/Cumulative Incidence Proportion

### 2.5. Diagnostic Yield of Different Genetic Tests Performed

Newborns who underwent genetic testing were reviewed for the type of genetic testing performed, including targeted variant testing, single gene testing, multi-gene panel testing, and whole exome sequencing (WES). The genetic testing results were categorized as pathogenic, variants of uncertain significance (VUSs), or negative. Cases with pathogenic variants were classified as solved. VUSs were classified as solved only when there was a strong phenotypic correlation, which refers to a clear and consistent association among the variant, the biochemical phenotype, and the observed clinical symptoms. Other cases with VUSs were classified as uncertain. Cases with negative results were classified as unsolved. The diagnostic yield of each genetic testing type was calculated using the following formula:

Diagnostic Yield (%)=(Number of Solved Cases/ Total Number of Cases)×100

This calculation was used descriptively to report diagnostic outcomes and testing trends. It was not intended to evaluate technical efficacy or the comparative performance of different testing platforms.

### 2.6. Statistical Analysis

The statistical analyses used Stata version 18 to categorize variables and examine associations among demographic characteristics, clinical characteristics, and metabolic disorders. We utilized chi-square, Fisher’s exact tests, and one-sample test of proportion for statistical comparisons. The cumulative incidence was calculated by dividing the number of cases by the total number of newborns screened. Exact 95% confidence intervals (CIs) were computed using the Poisson distribution and are expressed per 100,000 live births.

## 3. Results

### 3.1. Screening of Eligible Study Participants

**Figure 4:**
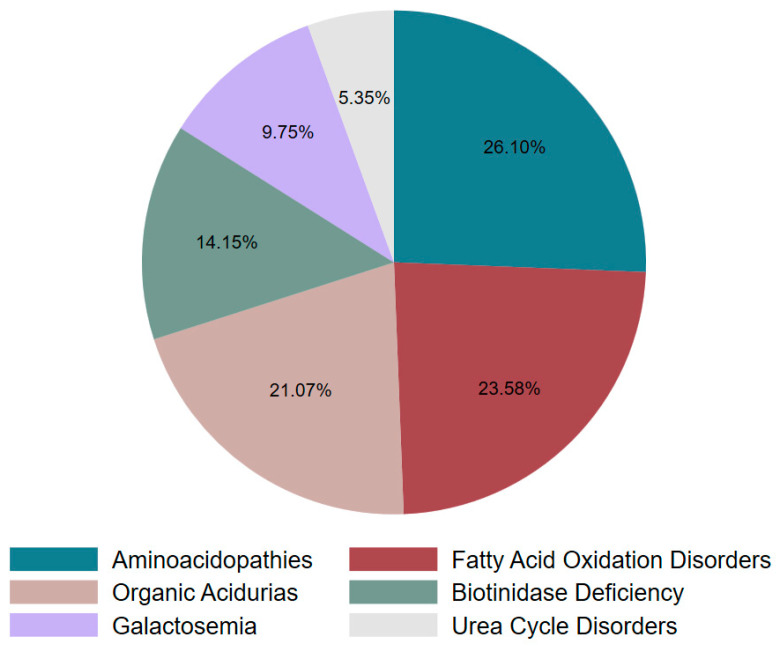
Categorical distribution of IEMs identified in our study.

As seen in Figure 3, the positive predictive value (PPV) of the overall screening program was 7.5%, calculated as the proportion of true positives (318) among all initial screen-positive cases (4219). Additionally, the PPV of referred cases was 59.7% (318/533), suggesting that nearly 60% of referred cases were confirmed with the disease after comprehensive evaluation. These 318 confirmed cases were further categorized into six groups: aminoacidopathies, organic acidurias, fatty acid oxidation disorders, urea cycle disorders, galactosemia, and biotinidase deficiency. Aminoacidopathies emerged as the most frequently reported category, constituting 26.10% of the cases (*n* = 83), as illustrated in Figure 4.

### 3.2. Demographics and Clinical Characteristics of Study Subjects

The demographic characteristics of the 318 cases with IEMs are provided in Table S1. There was almost equal distribution between genders: 49.1% females (*n* = 156) and 50.9% males (*n* = 162). Among these, 41.5% were Qatari (*n* = 132), forming the largest subgroup. The remaining cases, constituting 58.5% of the total, were of various non-Qatari ethnicities including Pakistanis 17.0% (*n* = 54), Egyptians 10.1% (*n* = 32), Indians 6.0% (*n* = 19), Syrians 4.7% (*n* = 15), Sudanese 4.4% (*n* = 14), Jordanians 3.1% (*n* = 10), and others 13.2% (*n* = 42), as illustrated in Figure S1.

In terms of gestational age (GA), the majority of cases (78.0%, *n*= 248) were born full-term (37 to 42 weeks), while late preterm (34 and 36 weeks) and preterm births (<34 weeks) accounted for 6.6% (*n* = 21) and 6.0% (*n* = 19) of the cases, respectively. GA was not recorded in 9.4% of cases (*n* = 30).

Consanguinity among parents was reported in 60.1% (*n* = 191) of cases, with first-cousin marriages being the most common (59.16%, *n* = 113). Non-consanguineous marriages accounted for 20.8% (*n* = 66), and information was not available for 19.2% (*n* = 61). In terms of family history of an IEM in the family, 34.9% (*n* = 111) of newborns had a positive family history, while 50.3% (*n* = 160) had a negative family history, which was unreported in 14.8% (*n* = 47) of cases (Table S1).

We also compared demographics, clinical characteristics, and the utilization of genetic testing between Qatari and non-Qatari populations (Table S2). Significant differences were observed in consanguinity, IEM family history, and the utilization of genetic testing. Consanguinity was more prevalent among Qataris (75.0%) compared to non-Qataris (49.5%). Likewise, a positive family history of IEM was more frequently reported among Qataris (56.8%) than non-Qataris (19.4%). There were also significant differences in the use of genetic testing: non-Qataris were less likely to undergo genetic testing (69.9% did not undergoing testing), compared to Qataris (20.5%). Among Qataris, targeted variant testing was more commonly performed (40.2%), while single gene testing was more performed among non-Qataris (15.6%).

**Figure 5:**
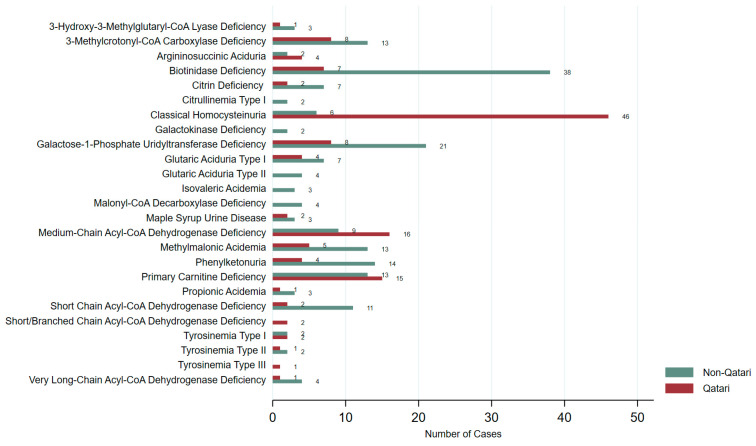
Distribution of IEMs identified in Qataris and non-Qataris in our study.

The distribution of identified IEMs also varied between groups (Figure 5). Classical Homocystinuria (HCU) and Medium-Chain Acyl-CoA Dehydrogenase Deficiency (MCAD) were more prevalent among Qataris, while Biotinidase deficiency, Galactose-1-Phosphate Uridylyltransferase (GALT) Deficiency, Phenylketonuria (PKU), and Short-Chain Acyl-CoA Dehydrogenase Deficiency (SCAD) were more frequently reported in non-Qataris. Figure S2 illustrates the distribution of IEMs across different ethnic groups, highlighting variation in disorder types among Qatar’s diverse population.

Following the categorization of the screened IEMs into the six disease groups, we conducted a detailed analysis of demographic and clinical characteristics across these categories: aminoacidopathies, organic acidemias, fatty acid oxidation disorders, urea cycle disorders, galactosemias, and biotinidase deficiency. Significant differences were observed in ethnicity, consanguinity, IEM family history, and types of genetic testing performed. Notably, aminoacidopathies were more prevalent in Qataris (67%, *n* = 56), while other categories were more frequent among non-Qataris. This group also showed the highest rates of consanguinity (75%) and positive family history (59%). Targeted genetic testing was more commonly conducted for the aminoacidopathies. Single gene testing was predominantly ordered for organic acidemias, whereas WES was primarily ordered for fatty acid oxidation disorders (see Table 1).

### 3.3. Calculated Incidence of Identified IEMs

Over 14 years, the number of newborns screened was 351,233 (Figure S3). The incidence of IEMs was calculated and presented as a cumulative incidence ratio (Table 2). HCU had the highest incidence within the cohort, as it was found to have an expected occurrence of 1 in every 6754 live births. This was followed by partial biotinidase deficiency with an incidence of approximately 1 in every 10,330 live births. Galactosemia due to GALT deficiency, PCD, MCAD, 3-MCC, and PKU had an accumulative incidence of 1:12,111, 1:12,544, 1:14,049, 1:16,725, and 1:19,513, respectively. GA-II and TYR1 showed the same incidence of 1 in every 87,808. The least frequently encountered IEMs CTLN1, TYR2, TYR3, and IVA with cumulative incidence ratios of 1:175,617, 1:117,078, 1:351,233, and 1:117,078, respectively. Overall, the incidence of the IEMs screened by the QNNSP yielded a total cumulative incidence of 1 in 1105 newborns.

### 3.4. Genetic Testing and Diagnostic Yield

Among the 318 cases with IEMs, 49.0% (*n* = 156) underwent genetic testing. Four types of genetic tests were identified: targeted variant testing, single gene testing, multi-gene panel testing, and WES (Figure S4). Of the 156 newborn cases genetically tested, 141 were confirmed, yielding an overall diagnostic rate of 90%.

**Figure 6:**
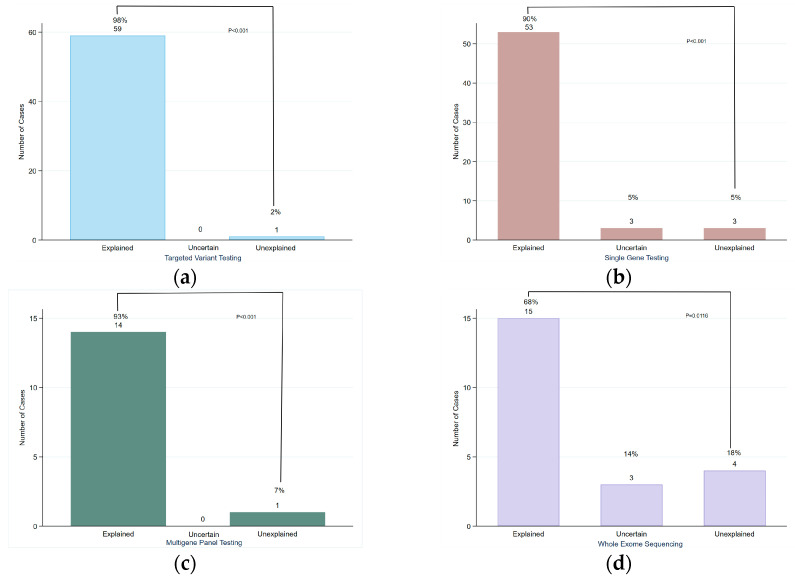
Diagnostic yield of different genetic tests performed: (**a**) targeted variant testing; (**b**) single gene testing; (**c**) multi-gene panel testing; (**d**) whole exome sequencing.

Familial targeted variant testing and single gene testing were the most commonly performed, each accounting for 38.5% (*n* = 60) and 37.8% (*n* = 59) of the cases, respectively. Targeted variant testing achieved the highest diagnostic yield, with 98% (*n* = 59) of cases solved, while single gene testing had a 90% (*n* = 53) diagnostic rate. Both showed statistically significant differences between solved and unsolved cases (*p* < 0.001). Multi-gene panel testing was performed in only 9.6% of the cases (*n* = 15). Notably, no uncertain cases were reported; however, three cases with variants of uncertain significance (VUS) were classified as solved due to the correlation between the variant detected and the phenotype. The difference between solved and unsolved cases was statistically significant (*p* < 0.001). WES was conducted in 14.1% of the newborns (*n* = 22) with a diagnostic yield of 68% (*n* = 15). This was significantly different from unsolved cases (18%, *n* = 4; *p* = 0.0116), as shown in Figure 6.

### 3.5. Genes and Genetic Findings for Screened IEMs

Table 3 describes the identified genetic variants associated with IEMs and their prevalence among both Qatari and non-Qatari cases in our cohort. We detected the known founder variant in the *CBS* gene (c.10006C > T) in all of the 37 Qatari newborns with HCU. Additionally, other variants such as c.1330G > A, c.409A > T, c.188C > T, and c.785C > T in the *CBS* gene were each reported in one non-Qatari newborn with HCU. Eight different variants in the *PAH* gene were identified among Qatari and non-Qatari cases with PKU, including p. Ala403Val, exon 3 deletion, p.Ala300Ser, c.169_171del, c.1199 G > C, c.293T > C, c.1184C > G, and c.1066-11G > A. Among PCD cases, 11 Qatari newborns were found to be homozygous for the pathogenic variant c.83G > T in the *SLC22A5* gene. Similarly, six Qatari newborns were found to be homozygous for the pathogenic variant c.362C > T in the *ACADM* gene known to be associated with MCAD diagnosis. In contrast, six Pakistani newborns were found to be homozygous for the pathogenic variant c.394C > T in the *MMACHC* gene associated with cobalamin C deficiency.

## 4. Discussion

### 4.1. Incidence

#### 4.1.1. IEM Incidence in Gulf Region

We have calculated the cumulative incidence of the 26 IEMs in Qatar to be 1 in 1105 newborns (95% CI: 1 in 989 to 1 in 1236). Based on an earlier study conducted in Qatar between December 2003 and July 2006, the cumulative incidence was found to be 1:1327 (95% CI: 1 in 917 to 1 in 2439) [1]. The increase in cumulative incidence suggests that either the prevalence of these metabolic disorders has increased over time, or the screening program has become more effective at detecting cases. However, the 95% confidence intervals for the two time periods overlap, suggesting that this difference is not statistically significant. Therefore, this apparent increase may reflect improved detection, expanded screening practices, or possibly a true rise in disease prevalence over time.

In a Saudi retrospective study from 2005 to 2012, the incidence of 1 in 1443 for 16 (IEMs) was reported [11], closely aligning with our study’s incidence of 1 in 1105 for 26 IEMs. Both studies screened a largely overlapping core panel of disorders, including PKU, MSUD, 3-MCC, MMA, GA-I, IVA, HMG, MCAD, galactosemia due to GALT deficiency, BTD, ASA, and CTLN1. However, our panel included additional conditions such as tyrosinemias (TYR1–3), other fatty acid oxidation disorders (e.g., VLCAD, SCAD, PCD), and further subtypes of galactosemia (GALK deficiency), which likely contribute to the higher total number of screened disorders. This suggests that inherited metabolic disorders are similarly prevalent in both countries, which could reflect common genetic background and founder variants in the region in addition to similar practices of cultural endogamy and consanguinity.

A study conducted in the United Arab Emirates (UAE) from 2011 to 2014 reported the incidence of 29 IEMs as 1 in 1787 live births [12]. This incidence is lower compared to the incidence reported in Saudi Arabia and in our study. In our study consanguinity was reported at 60.1%. Similarly in the UAE’s study, consanguineous marriages were identified as a major cause of IEMs, with 81.5% of affected families being consanguineous [12]. Their study demonstrated that effective premarital counseling and genetic screening are crucial components of genetic disease prevention programs [12]. By integrating premarital screening and NBS, it is expected that the incidence of IEMs can be significantly reduced. In Qatar, early detection and management of HCU through the premarital screening program was implemented in 2009 towards reducing HCU incidence [13].

#### 4.1.2. IEM Incidence in MENA Region

The incidence of IEM was reported in Libya to be 1 in 1458 live births [14]. Amino acid disorders and carbohydrate disorders were shown to be particularly common, accounting for 1 in 6158 and 1 in 6927 live births, respectively [14]. Their study found that 86.9% of the parents of the affected infants were consanguineous, which may account for the high prevalence of IEMs in Libya. As expected, a significant proportion of cases, 63.5%, had a family history of previously affected children, underscoring the genetic component of these conditions [14]. Family history was also observed in our study, comprising 34.9% of cases. In their study, amino acid disorders accounted for 25% of all IEM cases followed by carbohydrate disorders (14.9%), lysosomal storage diseases (14%), organic aciduria (9.3%), and energy metabolic deficiencies (9.3%) [14]. In comparison to our cohort, aminoacidopathies were also the most prevalent, constituting (26.1%) of the total cases, followed by fatty acid oxidation disorders (24.2%), then organic acidurias (20.4%). It seems that amino acid disorders are the most prevalent in both Qatar and the region.

A pilot study conducted in Egypt from 2008 to 2015 estimated the number of cases of IEMs in a hypothetical annual cohort of 2.5 million newborns to be 1286 based on the screening of seven specific IEMs among newborns [15]. It is projected that 40–50% of all cases detected were with PKU [15]. A prospective study at the Pediatrics and Neonatology Department of Sohag University Hospital in Egypt determined that among the included 308 neonates suspected to have an IEM, 30.2% were diagnosed with IEMs. In their study, the most frequently encountered diagnoses were PKU, GA-I, and MSUD [16]. The prevalent identifications included PKU (43%), GA-I (19.4%), and MSUD (14%) [16]. In our cohort, Egyptians were the third largest ethnicity (*n* = 32) after Qataris (*n* = 132), and among the Egyptians, PKU and BTD were the most frequently reported disorders. Moreover, 7 out of the 18 PKU cases were of Egyptian origin, reflecting the high incidence of PKU among Egyptians in our study as well. The variability in the incidence of genetic diseases among different Middle Eastern populations might be explained by founder effects [17].

#### 4.1.3. IME Incidence in Europe, USA, and China

A study in Italy found that the incidence of IEMs in the Italian pediatric population was 1 in 3707 live births [18]. While in the USA, the combined incidence of individual IEMs was estimated to be around 1 in 2500 live births [19]. In China, a nationwide survey using MS/MS for NBS reported an overall incidence of IEMs equivalent to 1 in 2585 births [20]. Similarly, a study in Taiwan reported an overall incidence of around 1 in 5882 live births [21]. These findings underscore the variability in incidence rates across different populations and geographical regions, underscoring the need for region-specific data to inform healthcare planning and resource allocation. We anticipate that these nations would exhibit lower incidence rates in contrast to the Gulf countries where consanguinity is high.

### 4.2. Genetic Testing

Of the 156 newborn cases genetically tested, 141 were confirmed, yielding an overall diagnostic rate of 90%. Although genetic testing was made available to all patients within the framework of their clinical management, not all individuals opted to undergo testing due to factors such as financial constraints, apprehensions regarding stigma, and individual preferences. Consequently, the cohort that underwent testing may not be fully representative of the broader patient population, introducing a potential selection bias that could affect the generalizability and interpretation of the diagnostic yield.

The reason for targeted variant testing and single gene testing to have the highest number of cases for IEMs stems from the genetic makeup of metabolic disorders, which are usually monogenic [22]. Because of this, the appropriate testing options would be either single gene testing or targeted variant testing in cases of known founder/familial variants [19,23]. In non-Qatari cases, testing for the founder variant in Qatar would not be a suitable option, instead, full gene sequencing would be preferred. For example, for HCU, targeted testing for the known Qatari-variant p.R336C (c.1006C > T) in the *CBS* gene was the most utilized test for Qataris. However, for non-Qatari cases, the testing option was full *CBS* gene sequencing.

Salman et al. (2022) found that a majority of the requests (53%) were for single gene testing, while 36% were for WES, and 10% were for gene panels [24]. The overall diagnostic yield for genetic testing of IEMs was 64.3%. Single gene testing had a diagnostic yield of 75%, while WES had a diagnostic yield of 49% for complex cases like mitochondrial disorders [24]. In light of WES, our diagnostic yield was 68%. In comparison, a study conducted in France demonstrated that WES had a diagnostic yield of 64% for metabolic disorders in pediatric patients, indicating its effectiveness in diagnosing a broader range of genetic conditions [25]. This emphasizes the importance of selecting the appropriate genetic testing method based on the suspected disorder and the clinical context.

### 4.3. Genetic Findings

HCU has been identified as a prevalent metabolic disease in Qatar, with the highest known incidence globally. In our study, the cumulative incidence of HCU was 1:6754, calculated out of the 52 HCU newborn cases identified, from the total number of 351,233 newborns screened between 2010 and 2023. A previous study conducted by Zschocke et al. 2009 reported an incidence of 1:1800 considering Qatari neonates only, in which 7 had HCU out of 12,603 screened Qatari neonates in a two year span [26], surpassing the worldwide range of 1:200,000 to 1:350,000 live births [26,27]. The high incidence of HCU observed among the Qatari population is largely attributed to consanguinity (which is estimated at 66.2%) [28] and a founder variant, with a carrier frequency of approximately 2% [29]. In our study, we identified the known founder variant (*CBS*: c.10006C > T) in all of the 37 Qatari newborns with HCU in our cohort, reflecting the impact of founder variant and autozygosity in increasing the disease incidence.

Another interesting variant that was identified in our study is the c.83G > T pathogenic variant in the *SLC22A5* gene, which was reported in 11 Qatari newborns with PCD. This indicates that this variant may be prevalent in Qataris. The c.83G > T variant in the *SLC22A5* gene is a missense variant that causes the substitution of arginine with leucine at position 28 of the SLC22A5 protein. This alteration can interfere with the normal function of the SLC22A5 protein, which is responsible for transporting carnitine into cells, thus having reduced carnitine uptake, leading to the characteristic features of primary carnitine deficiency [30]. Another variant that seems to be prevalent in Qataris is the pathogenic variants (c.362C > T) in the *ACADM* gene known to be associated with MCAD diagnosis, which was detected in six Qataris in homozygous state.

Our study reported six Pakistani newborns with the same homozygous pathogenic variant c.394C > T in the *MMACHC* gene associated with cobalamin C deficiency. Homozygous pathogenic variant c.394C > T in the *MMACHC* gene has been detected in patients who are Bengali and Pakistani in origin [31]. This variant may be a potential founder variant in these countries [31]. Moreover, patients of Indian, Pakistani, or Middle Eastern heritage have been found to have a late-onset cobalamin C deficiency linked to this particular variant, highlighting its significance in disease manifestation within these ethnic groups [32].

Further studies specifically on the pathogenic variants c.83G > T in the *SLC22A5* gene associated with primary carnitine deficiency and c.350C > T in the *ACADM* gene associated with MCAD in Qatar would be beneficial to comprehensively understand their role and frequencies in the population and to investigate the potential to include them in premarital screening.

The study’s retrospective nature inherently carries the risk of missing records and inaccuracies. In addition, the study may be affected by the evolution of diagnostic criteria over the studied period and the sensitivity and specificity of different genetic tests employed may influence the diagnostic yield. Moreover, the variability across various newborn screening programs in different countries in terms of the tests or conditions performed as well as the various study designs hinders accurate comparison.

## 5. Conclusions

Our study provides the incidence of IEMs in Qatar over a 14-year interval. The overall incidence of the 26 IEMs studied is 1:1105, which is significantly high compared to other outbred populations, but seems relatively similar to reports from other Gulf countries. Our study highlighted the significantly elevated incidence of HCU, attributed to the founder variant p. Arg336Cys in the Qatari population. Considering IEM groups, aminoacidopathies were the most prevalent, followed by fatty acid oxidation disorders, organic acids, biotinidase deficiency, and lastly urea cycle disorders. The calculated incidences of these disorders provide valuable insights into their prevalence in the population, highlighting the importance of NBS programs for the early detection and management of such conditions. In our study, the overall diagnostic yield of genetic testing was 90%. Our study serves as a foundation to help policymakers develop strategies to further reduce the incidence of IEMs in Qatar. It will facilitate improving IEM prevention strategies, for example, through premarital screening and Preimplantation Genetic Testing for Monogenic Disorders.

## Figures and Tables

**Table 1:** Correlation of metabolic disorder groups with demographic and clinical characteristics.

		Aminoacidopathies	Organic Acidemias	Fatty Acid Oxidation Disorders	Urea Cycle Disorders	Galactosemia	Biotinidase Deficiency	*p*-Value
		*n* = 83 (%)	*n* = 67 (%)	*n* = 75 (%)	*n* = 17 (%)	*n* = 31 (%)	*n* = 45 (%)	
Gender	Female	35 (42%)	33 (49%)	35 (47%)	10 (59%)	19 (61%)	24 (53%)	0.48
	Male	48 (58%)	34 (51%)	40 (53%)	7 (41%)	12 (39%)	21 (47%)	
Ethnicity	Non-Qatari	27 (33%)	46 (69%)	41 (55%)	11 (65%)	23 (74%)	38 (84%)	<0.001
	Qatari	56 (67%)	21 (31%)	34 (45%)	6 (35%)	8(26%)	7 (16%)	
Consanguinity	Yes	62 (75%)	43 (64%)	45 (60%)	7 (41%)	17 (55%)	17 (38%)	0.003
	No	8 (10%)	13 (19%)	16 (21%)	4 (24%)	6 (19%)	19 (42%)	
	Not Reported	13 (16%)	11 (16%)	14 (19%)	6 (35%)	8 (26%)	9 (20%)	
IEM Family	Positive	49 (59%)	18 (27%)	30 (40%)	1 (6%)	10 (32%)	3 (7%)	<0.001
History	Negative	25 (30%)	40 (60%)	36 (48%)	11 (65%)	16 (52%)	32 (71%)	
	Not Reported	9 (11%)	9 (13%)	9 (12%)	5 (29%)	5(16%)	10 (22%)	
Genetic Testing	Targeted Variant Testing	38 (46%)	8 (12%)	12 (16%)	0 (0%)	1 (3%)	1 (2%)	<0.001
	Single Gene Testing	11 (13%)	22 (33%)	16 (21%)	1 (6%)	4 (13%)	5 (11%)	
	Multi-gene Panel Testing	4 (5%)	5 (7%)	2 (3%)	2 (12%)	1 (3%)	1 (2%)	
	WES	2 (2%)	6 (9%)	9 (12%)	1 (6%)	3 (10%)	1 (2%)	
	None	28 (34%)	25 (37%)	34 (45%)	13 (76%)	21 (68%)	36 (80%)	
	Not Reported	0 (0%)	1 (1%)	2 (3%)	0 (0%)	1 (3%)	1 (2%)	

**Table 2:** Distribution of detected cases and incidence of each screened disorder.

Category	No	Disease	No. of Cases	Cumulative Incidence Ratio	Incidence per 100,000 (95% CI)
Aminoacidopathies	1	HCU	52	1:6754	14.8 (11.1–19.4)
	2	PKU	18	1:19,513	5.1 (3.0–8.1)
	3	MSUD	5	1:70,247	1.4 (0.5–3.3)
	4	TYR1	4	1:87,808	1.1 (0.3–2.9)
	5	TYR2	3	1:117,078	0.9 (0.2–2.5)
	6	TYR3	1	1:351,233	0.3 (0.0–1.6)
Organic Acidemias	7	3-MCC	21	1:16,725	6.0 (3.7–9.1)
	8	MMA	18	1:19,513	5.1 (3.0–8.1)
	9	GA-I	11	1:31,930	3.1 (1.6–5.6)
	10	PA	4	1:87,808	1.1 (0.3–2.9)
	11	HMG	4	1:87,808	1.1 (0.3–2.9)
	12	MCD	4	1:87,808	1.1 (0.3–2.9)
	13	IVA	3	1:117,078	0.9 (0.2–2.5)
	14	SBCAD	2	1:175,617	0.6 (0.1–2.1)
Fatty Acid Oxidation Disorders	15	PCD	28	1:12,544	8.0 (5.3–11.5)
	16	MCAD	25	1:14,049	7.1 (4.6–10.5)
	17	SCAD	13	1:27,018	3.7 (2.0–6.3)
	18	VLCAD	5	1:70,247	1.4 (0.5–3.3)
	19	GA-II	4	1:87,808	1.1 (0.3–2.9)
Urea Cycle Disorders	20	Citrin Deficiency	9	1:39,026	2.6 (1.2–4.9)
	21	ASA	6	1:58,539	1.7 (0.6–3.7)
	22	CTLN1	2	1:175,617	0.6 (0.1–2.1)
Galactosemias	23	Galactosemia due to GALT Deficiency	29	1:12,111	8.3 (5.5–11.9)
	24	Galactosemia due to GALK Deficiency	2	1:175,617	0.6 (0.1–2.1)
Biotinidase Deficiency	25	Partial BTD	34	1:10,330	9.7 (6.7–13.5)
	26	Profound BTD	11	1:31,930	3.1 (1.6–5.6)
		BTD Combined	45	1:7805	12.8 (9.3–17.1)
		Total	318	1:1105	90.5 (80.9–101.1)

Abbreviations: HCU—Classical Homocystinuria; PKU—Phenylketonuria; MSUD—Maple Syrup Urine Disease; TYR1—Tyrosinemia Type I; TYR2—Tyrosinemia Type II; TYR3—Tyrosinemia Type III; 3-MCC—3-Methylcrotonyl-CoA Carboxylase Deficiency; MMA—Methylmalonic Acidemia; GA-I—Glutaric Acidemia Type I; PA—Propionic Acidemia; HMG—3-Hydroxy-3-Methylglutaryl-CoA Lyase Deficiency; MCD—Malonyl-CoA Decarboxylase Deficiency; IVA—Isovaleric Acidemia; PCD—Primary Carnitine Deficiency; MCAD—Medium-Chain Acyl-CoA Dehydrogenase Deficiency; SCAD—Short-Chain Acyl-CoA Dehydrogenase Deficiency; VLCAD—Very-Long-Chain Acyl-CoA Dehydrogenase Deficiency; GA-II—Glutaric Acidemia Type II; SBCAD—Short/Branched-Chain Acyl-CoA Dehydrogenase Deficiency; ASA—Argininosuccinic Aciduria; CTLN1—Citrullinemia Type I; GALT Deficiency—Galactose-1-Phosphate Uridylyltransferase Deficiency; GALK Deficiency—Galactokinase Deficiency; Partial BTD—Partial Biotinidase Deficiency; Profound BTD—Profound Biotinidase Deficiency.

**Table 3:** Genes and variants identified in cases who underwent genetic testing.

Disease	Gene	cDNA Change	Amino Acid Change	Zygosity	Variant Classification ACMG	Phenotype Correlation	Qatari	Non-Qatari
**HCU**	*CBS*	c.1006C > T	p.Arg336Cys	Homo	PV	Solved	37	2
		c.1330G > A	p.Asp444Asn	Homo	PV	Solved	0	1
		c.409A > T	p.Lys137 *	Homo	PV	Solved	0	1
		c.188C > T	p.Ser63Phe	Homo	LPV	Solved	0	1
		c.785C > T	p.Thr262Met	Homo	PV	Solved	0	1
**PKU**	*PAH*	c.1208C > T	p.Ala403Val	CH	PV	Solved	0	1
		Exon 3 Deletion	_		PV			
		c.898G > T	p.Ala300Ser	Homo	PV	Solved	1	0
		c.169_171del	p.Asp57del	Homo	PV	Solved	0	1
		c.1199 G > C	p.Arg400Thr	Homo	PV	Solved	1	0
		c.1066 − 11G > A	_	Homo	PV	Solved	0	1
		c.293T > C	p.Leu98Ser	Homo	PV	Solved	1	0
		c.1184C > G	p.Ala395Gly	Homo	PV	Solved	1	0
**ASA**	*ASL*	c.375G > T	p.Met125Ile	Homo	LPV	Solved	1	0
		c.299T > C	p.Ile100Thr	Homo	PV	Solved	1	0
**BTD**	*BTD*	c.1367A > G	p.Tyr456Cys	Homo	PV	Solved	0	1
		c.89C > T	p.Leu30Pro	Homo	VUS	Solved	1	0
		c.1432G > C	p.Ala478Pro	CH	LPV	Solved	0	1
		c.968A > G	p.His323Arg					
		c.497G > A	p.Cys166Tyr	CH	PV	Solved	2	0
		c.1270G > C	p.Asp424His					
		c.968A > G	p.His323Arg	CH	LPV	Solved	1	0
		c.1368A > C	p.Gln456His					
		c.1330G > C	p.Asp444His	Homo	VUS	Solved	0	1
		c.1259G > C	p.Cys420Ser	Homo	LPV	Solved	0	1
**PCD**	*SLC22A5*	c.83G > T	p.Ser28Ile	Homo	PV	Solved	11	1
		c.371 A > G	p.Tyr124Cys	Homo	VUS	Uncertain	0	1
		c.391G > A	p.Glu131Lys	CH	VUS	Uncertain	2	0
		C.83G > T	p.Ser28Ile		PV			
**CTLN1**	*ASS1*	c.598 − 2A > G	IVS9 − 2A > G	Homo	PV	Solved	0	1
**GA-I**	*GCDH*	c.1133C > T	p.Ala378Val	Homo	VUS	Solved	0	1
		c.706 T > C	p.Phe236Lys	Homo	PV	Solved	2	0
		c.671T > G	p.Val224Gly	*	VUS	Unsolved	0	1
		c.742C > T	p.Pro248Ser	Homo	PV	Solved	0	2
		c.505 + 1G > A	_	CH	PV	Solved	0	1
		c.1147C > T	p.Arg383Cys		LPV			
		c.756C > T	p.Gly252	Homo	VUS	Solved	1	0
**GALK**	*GALK1*	c.853_874del22	p.1285RfsX4	Homo	PV	Solved	0	1
**GALT**	*GALT*	c.563A > G	p.Gln188Arg	Homo	PV	Solved	0	1
		c.772C > T	p.Arg258Cys	Homo	PV	Solved	1	0
		c.247G > A	p.Gly83Arg	Homo	VUS	Uncertain	1	0
		c.299C > G	p.Pro100Arg	CH	LPV	Solved	0	1
		c.940A > G	p.Asn314Asp		PV			
		c.563A > G	p.Gln188Arg	CH	PV	Solved	1	0
		c.-119_-116del	_					
		c.940A > G	p.Asn314Asp					
		c.1049C > T	p.T350I	CH	VUS	Solved	1	0
		c.-119_-116del	p.?		PV			
		VUS in Exon 2	-	CH	VUS	Uncertain	0	1
		c.378 − 27G > C, c.508 − 24G > A, c.507 + 62G > A						
		c.940A > G	p.Asn314Asp					
**HMG**	*HMGCL*	c.206_207delCT	p.Ser69CysfsX11	Homo	PV	Solved	0	2
		c.914_915delTT	p.Phe305TyrfsX10	Homo	PV	Solved	1	0
**GA-II**	*ETFB*	c.491G > A	p.Arg164Gln	Homo	LPV	Solved	0	2
**MCAD**	*ACADM*	c.362C > T	p.Thr121Ile	Homo	PV	Solved	6	0
		c.329A > G	p.Glu110Gly	CH	LPV	Solved	3	0
		c.362C > T	p.Thr121Ile		PV			
		c.984delG	p.M328IfsX5	CH	PV	Solved	0	1
		c.653C > G	p.A218G		VUS			
		c.572G > A	p.W191X	Homo	PV	Solved	1	0
		c.799G > A	p.G267R	Homo	PV	Solved	0	1
		c.671G > A	p.Trp224 *	Homo	LPV	Solved	1	0
		c.374C > T	p.Thr125IIe	Homo	PV	Solved	1	0
**3-MCC**	*MCCC2*	_	partial gene del including exon 13	Homo	PV	Solved	2	0
		c.538C > T	p.Arg180Ter	Homo	PV	Solved	3	0
		c.1150_1216del67	_	Homo	PV	Solved	1	0
**MMA**	*MMUT*	c.1462G > A	p.Gly488Arg	CH	VUS	Solved	0	1
		PV						
		c.571G > A	p.A191T	Homo	LPV	Solved	2	0
		c.1463G > T	p.Gly488Val	Homo	VUS	Solved	1	0
	*MMAA*	c.489delT	p.Phe163LeufsX15	Homo	PV	Solved	0	1
	*MMAB*	c.571C > T	p.R191W	Homo	PV	Solved	1	0
	*MMACHC*	c.394C > T	p.Arg132 *	Homo	PV	Solved	0	6
		c.616del	p.Arg206Glyfs *4	Homo	LPV	Solved	1	0
		c.271dup	p.Arg91Lysfs *14	Homo	PV	Solved	0	1
		c.331C > T	p.Arg111 *	Homo	PV	Solved	0	1
**MCD**	*MLYCD*	c.1213dup	p.Tyr405Leufs *74	Homo	LPV	Solved	0	2
		c.1A > C	p.Met1?	Homo	LPV	Solved	0	1
**PA**	*PCCA*	c.2062delT	p.C688VfsX2	Homo	LPV	Solved	0	1
		c.425 G > A	p.Gly142Asp	Homo	PV	Solved	1	0
**SCAD**	*ACADS*	c.136 C > T	p.Arg46Trp	Homo	LPV	Solved	0	1
		c.796 − 3C > G	IVS6 − 3C > G	Homo	VUS	Uncertain	1	0
**SBCAD**	*ACADSB*	c.303 + 3A > G	_	Homo	PV	Solved	2	0
**TYR1**	*FAH*	c.1A > G	p.M1	Homo	PV	Solved	1	0
**TYR2**	*TAT*	c.169C > T	p.R57X	Homo	PV	Solved	0	1
		c.839A > C	p.K280T	Homo	VUS	Solved	1	0
		c.1297C > T	p.R433W	Homo	LPV	Solved	0	1
**TYR3**	*HPD*	c.85G > A	p.A29T	Homo	VUS	Solved	1	0
**VLCAD**	*ACADVL*	c.1835_1860delinsG	p.A612Gfs *60	Homo	LPV	Solved	0	1
		c.65 C > A	p.S22 *	Homo	PV	Solved	1	0
		c.1246_1248del	p.A416del	CH	VUS	Solved	0	1
		c.1829 C > T	p.A610V					

Note: Homo: Homozygous; CH: Compound Heterozygous; VUS: Variant of Uncertain Significance; PV: Pathogenic Variant; LPV: Likely Pathogenic Variant; Variant Classification: By GeneDx that is following ACMG criteria. * In this case, biochemical testing confirmed the diagnosis; however, molecular testing was limited in detecting the second variant.

## Data Availability

Data supporting the findings of this study are available from the corresponding author upon reasonable request due to legal and ethical considerations.

## Supplementary Materials

The following supporting information can be downloaded at: https://www.mdpi.com/article/10.3390/ijns11030050/s1. Table S1: Demographic and characteristics of 318 cases studied; Figure S1: Frequency distribution of IEM cases by ethnicity; Figure S2: Distribution of specific IEM disorder types by ethnicity; Table S2: Association between the two population groups (Qataris and non-Qataris) with demographic and clinical characteristics; Figure S3: Number of screened newborns through NBS in Qatar from 2010 to 2023; Figure S4. Diagnostic yield of four genetic tests performed on included newborns.

## Abbreviations

The following abbreviations are used in this manuscript:

NBS	Newborn Screening
IEMs	Inborn Errors of Metabolism
QNNSP	Qatar National Newborn Screening Program
HCU	Classical Homocystinuria
MS/MS	Mass Spectrometry
ACMG	American College of Medical Genetics and Genomics
VUSs	Variants of Uncertain Significance
PGT-M	Preimplantation Genetic Testing for Monogenic Disorders
PKU	Phenylketonuria
MSUD	Maple Syrup Urine Disease
TYR1	Tyrosinemia Type I
TYR2	Tyrosinemia Type II
TYR3	Tyrosinemia Type III
3-MCC	3-Methylcrotonyl-CoA Carboxylase Deficiency
MMA	Methylmalonic Acidemia
GA-I	Glutaric Acidemia Type I
PA	Propionic Acidemia
HMG	3-Hydroxy-3-Methylglutaryl-CoA Lyase Deficiency
MCD	Malonyl-CoA Decarboxylase Deficiency
IVA	Isovaleric Acidemia
PCD	Primary Carnitine Deficiency
MCAD	Medium-Chain Acyl-CoA Dehydrogenase Deficiency
SCAD	Short-Chain Acyl-CoA Dehydrogenase Deficiency
VLCAD	Very-Long-Chain Acyl-CoA Dehydrogenase Deficiency
GA-II	Glutaric Acidemia Type II
SBCAD	Short/Branched-Chain Acyl-CoA Dehydrogenase Deficiency
ASA	Argininosuccinic Aciduria
CTLN1	Citrullinemia Type I
GALT	Galactose-1-Phosphate Uridylyltransferase Deficiency
GALK	Galactokinase Deficiency
BTD	Biotinidase Deficiency
