# Supplementary material for: Qatar’s National Expanded Metabolic Newborn Screening Program: Incidence and Outcomes
Source: Int J Neonatal Screen. 2025 Jun 30;11(3):50. doi: 10.3390/ijns11030050 (PMC12285972; doi:10.3390/ijns11030050)
Supplement: Supplementary file 1 [file IJNS-11-00050-s001.zip › IJNS-3626574-supplementary.pdf]

Supplementary Material:

Table S1. Demographic and characteristics of the 318 cases studied

**Demographics Characteristics**	**Total N= 318 (%)**
**Gender**	
Female	156 (49.1%)
Male	162 (50.9%)
**Ethnicity**	
Qatari	132 (41.5%)
Non-Qatari (27 Ethnicities)	186 (58.5%)
**Gestational Age**	
Full-term	248 (78.0%)
Late preterm	21 (6.6%)
Preterm	19 (6.0%)
Not reported	30 (9.4%)
**Consanguinity**	
Yes	191 (60.1%)
No	66 (20.8%)
Not reported	61 (19.2%)
**Family History**	
Positive	111 (34.9%)
Negative	160 (50.3%)
Not reported	47 (14.8%)

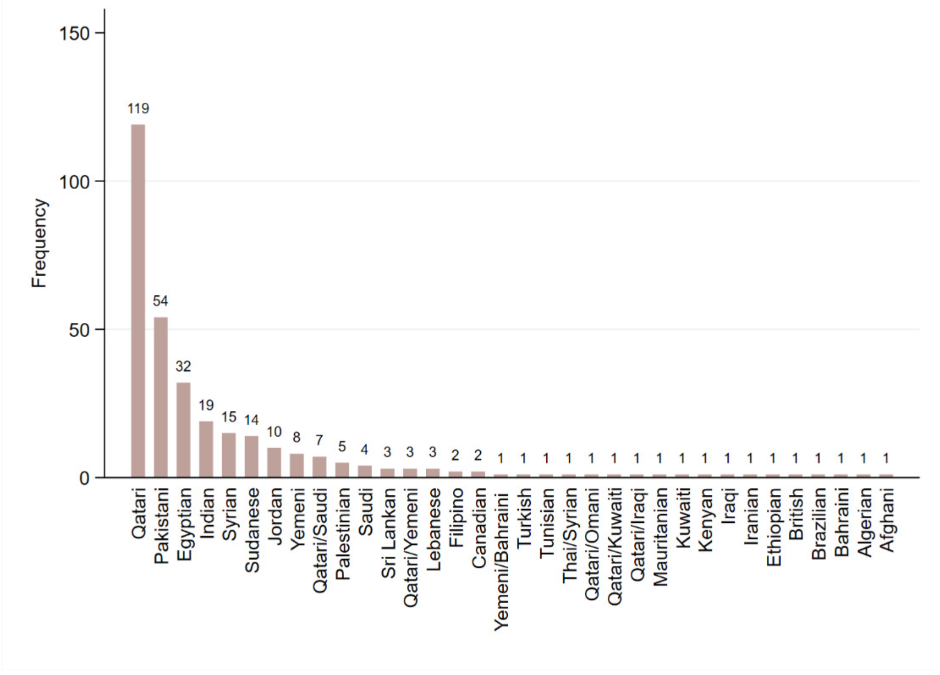

Figure S1. Frequency distribution of IEM cases by ethnicity

As of 2023, Qatar's total population was ~2.9 million, with Qatari nationals accounting for 11.6% and expatriates accounting for 88.4%. Although IEM cases were higher among non-Qataris (186 cases) compared to Qataris (132 cases), we analyzed these case counts relative to the underlying population structure. When adjusted for population size, the incidence of IEMs was 38.8 per 100,000 among Qataris and 7.1 per 100,000 among non-Qataris. These findings suggest a higher burden of IEMs among the Qatari population.

Worldometer. (2023). *Qatar population (live)*. Retrieved from https://www.worldometers.info/world-population/qatar-population/

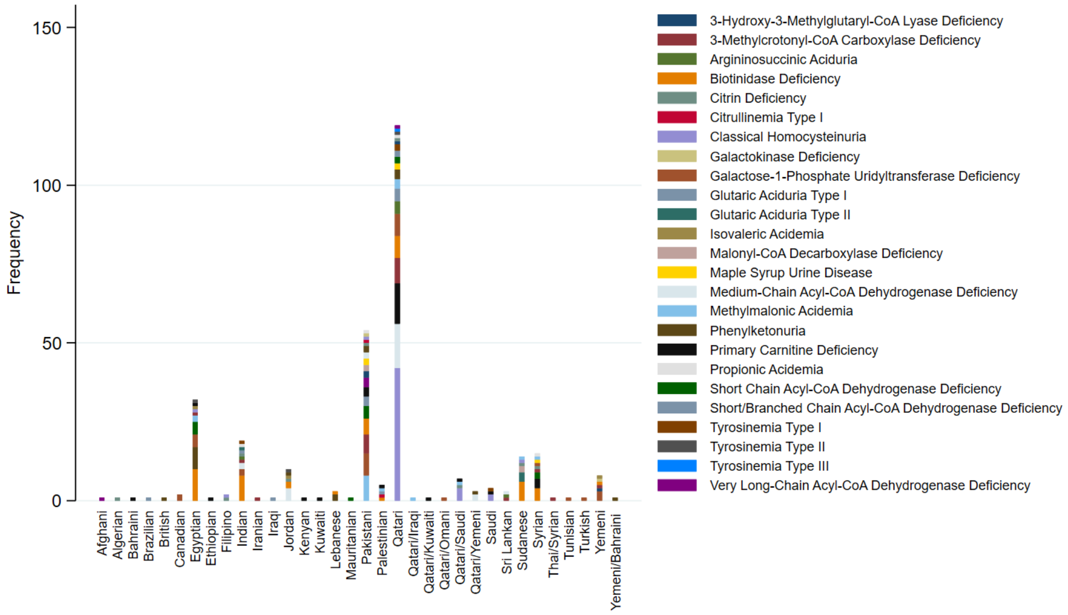

Figure S2. Distribution of specific IEM disorder types by ethnicity

Table S2. Association between the two population groups (Qataris and non-Qataris) with demographic and clinical characteristics

**Characteristics**		**Non-Qatari N=186 (%)**	**Qatari N=132 (%)**	**p-value**
Consanguinity	Yes	92 (49.5%)	99 (75.0%)	<0.001
	No	53 (28.5%)	13 (9.8%)	
	Not reported	41 (22.0%)	20 (15.2%)	
Family History	Positive	36 (19.4%)	75 (56.8%)	<0.001
	Negative	119 (64.0%)	41 (31.1%)	
	Not reported	31 (16.7%)	16 (12.1%)	
Genetic Testing	Targeted Variant Testing	7 (3.8%)	53 (40.2%)	<0.001
	Single Gene Testing	29 (15.6%)	30 (22.7%)	
	Multigene Panel Testing	5 (2.7%)	10 (7.6%)	
	WES	13 (7.0%)	9 (6.8%)	
	None	130 (69.9%)	27 (20.5%)	
	Not Reported	2 (1.1%)	3 (2.3%)	

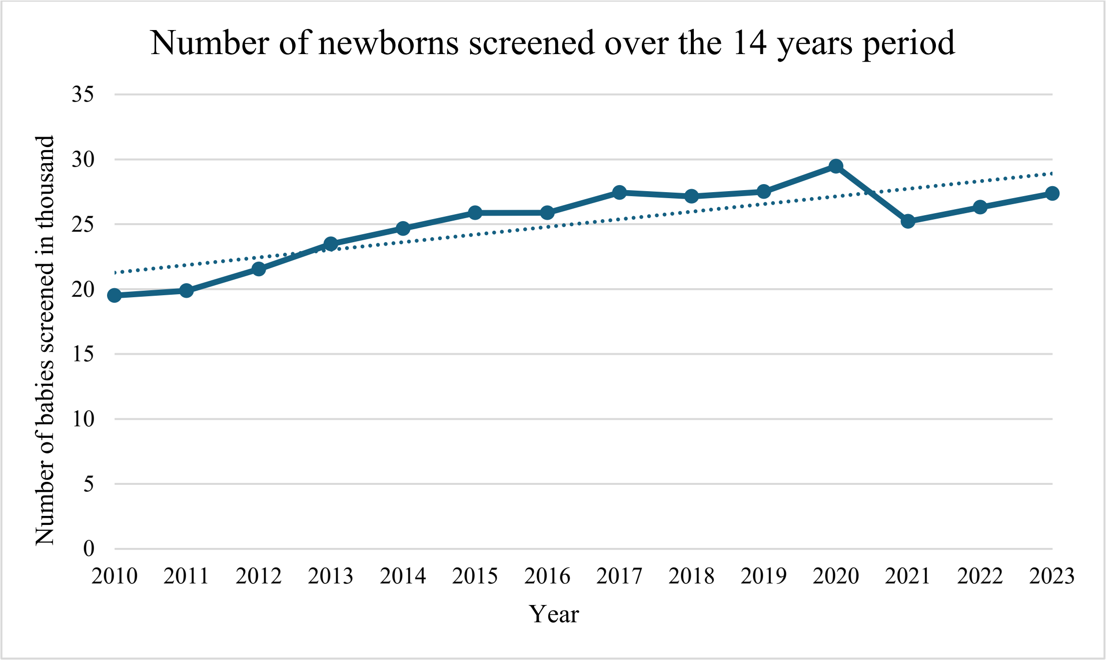

Figure S3. Number of screened newborns through NBS in Qatar from 2010-2023

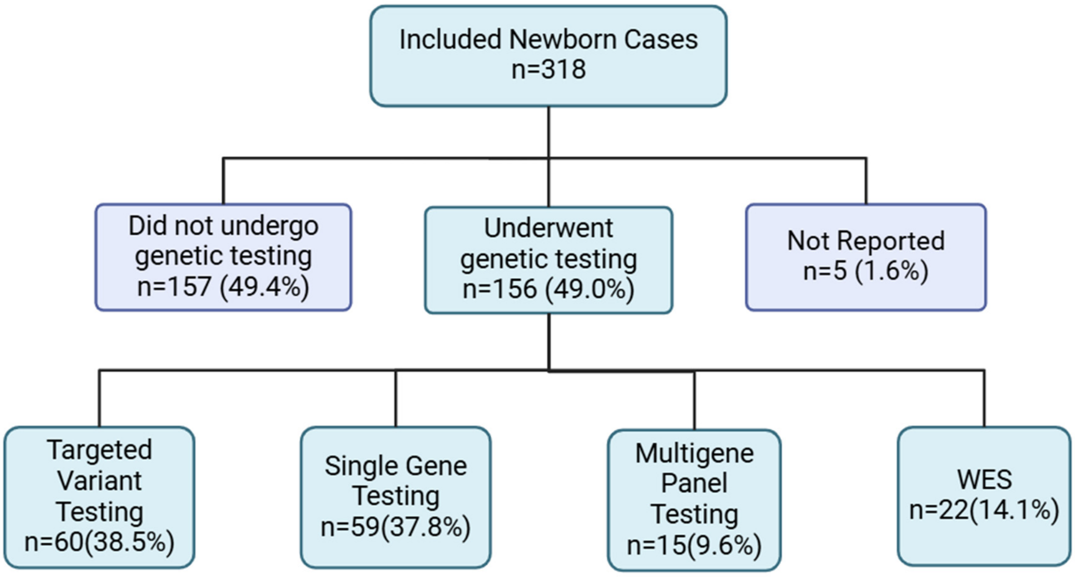

Figure S4. Diagnostic yield of the four genetic tests performed among included newborns
